# Inclusion Body Expression and Refolding of Recombinant Bone Morphogenetic Protein-2

**Published:** 2018

**Authors:** Davood Nasrabadi, Siamak Rezaeiani, Ali Sayadmanesh, Mohamadreza Baghaban Eslaminejad, Aliakbar Shabani

**Affiliations:** 1. Department of Medical Biotechnology, Semnan University of Medical Sciences, Semnan, Iran; 2. Department of Stem Cell and Developmental Biology, Cell Science Research Center, Royan Institute for Stem Cell Biology and Technology, ACECR, Tehran, Iran; 3. Student Research Committee, Semnan University of Medical Sciences, Semnan, Iran; 4. Department of Molecular Systems Biology, Cell Science Research Center, Royan Institute for Stem Cell Biology and Technology, ACECR, Tehran, Iran

**Keywords:** Bone morphogenetic protein-2, Cloning, *Escherichia coli*, Inclusion bodies, Protein refolding

## Abstract

**Background:**

Bone Morphogenetic Protein-2 (BMP-2) is a cysteine rich growth factor expressed in homodimeric form and has a pivotal role in osteochondral development and fracture healing. Recent studies have benefited more from recombinant BMP-2 in osteochondral tissue engineering. Cost-effective and easy production at large scale makes *Escherichia coli* (*E. coli*) the first choice for recombinant protein expression programs. However, inclusion body aggregation and refolding process limits production and purification of recombinant BMP-2 in bacterial systems.

**Methods:**

BMP-2 encoded gene was optimized for expression in bacterial expression system and synthesized with proper restriction sites. The optimized sequence was then cloned in a pET28a expression vector and expressed in Origami^™^ *E. coli* strain. The aggregated and monomeric BMP-2 was refolded and purified comparing two oxidoreductase systems and refolding methods as well as different purification techniques. The biological activity of recombinant protein was investigated by increasing alkaline phosphatase activity (ALK) of ATDC-5 cell line.

**Results:**

No difference was observed between oxidoreductase systems in improving the efficiency of protein refolding. However, comparisons between two refolding methods showed that pooling monomeric BMP-2 that was refolded under mild condition with equal volume of it refolded under severe oxidoreductase condition resulted in production of more active dimeric protein.

**Conclusion:**

A new method for production of biologically active dimeric form of BMP-2 in *E. coli* expression system was established in this study.

## Introduction

Bone morphogenetic proteins belong to transforming growth factor β superfamily which include TGF β, Activin, Nodals, Inhibins and Growth Differentiation Factors (GDFs) family. The members of TGF β superfamily have a vital role in embryonic development 1 and sending messages through smad signaling pathway 2. Over 20 BPM members have been identified to date 3, among them BMP-2, BMP-3, BMP-4 and BMP-7 have been studied more than any others. BMP-2 is a cysteine rich protein that plays an important role in many stages of chondrocytes differentiation and maturation rather than osteogenesis *in vivo*. The mature BMP-2 is a disulfide -connected homo-dimer, composed of 114 amino acids derived from the C-terminal sequence of a 396 amino acid precursor polypeptide. The intra and inter chain disulfide bonds are essential for active structure formation and ligand binding to cell surface receptors.

Cysteine rich heterologous protein production in prokaryotic systems leads to inclusion body formation and protein aggregation 4,5. Since they are enclosed in inclusion bodies, purification of aggregated proteins is rather a convenient process while their refolding is a laborious and time-consuming procedure.

TGF beta family proteins have been proposed to have multiple beta sheets and hydrophobic structures, as they can minimize water solubility and induce improper protein refolding patterns 6.

Protein refolding procedure generally begins with aggregated protein extraction and dissolving in high concentration chaotropic agents such as urea and guanidine hydrochloride (GuHCl). Chaotropic interaction unbraids entanglement structure of proteins and dissolves aggregated proteins in water. Afterwards, urea or GuHCl is replaced with additives or surfactants such as L-Arginine 7,8, L-proline 9, Glycerol 10 or detergents such as CHAPS 11, in order to prevent precipitation in the next stage of protein refolding process.

Generally, protein refolding method is an experimental technique-typically with less than 50% efficiency- and is highly dependent on protein type, refolding buffer compounds and additives.

One of the first and comprehensive studies on BMP-2 refolding was done by Vallejo 12. He showed that the refolding yield is particularly sensitive to protein concentration, temperature, pH, and the presence of aggregation inhibitors. However, the overall refolding yield obtained by him was 38% by optimizing the conditions for all parameters.

Refolded BMP-2 protein had the ability to induce osteogenesis *in vivo* when Sharapova *et al* applied it in a model of ectopic osteogenesis 13. They expressed and purified BMP-2 from *Escherichia coli (E. coli)* BL21 (DE3) inclusion body and refolded it using a buffer lacking a proper redox conditions. Although in previous reports the effects of pH 14 have been studied well enough to evaluate the refolding efficiency and biological activity of BMP-2, the influence of kind and quantity of different oxidoreductase agents on refolding efficiency has not been well established yet.

In this study, different oxidoreductase system effects on protein homodimeric formation procedure were compared and a simple method for protein dimer purification was established in addition to evaluation of the effects of changes in refolding process on protein activity.

## Materials and Methods

### Gene synthesis and cloning

Protein coding sequence of 114 N -terminal amino acid chain for bone morphogenetic protein2 (UniProt-KB-P12643) was retrieved from genbank |NM_001200.2| and optimized for expression in Origami 2(DE3) pLys strain of *E. coli* (Novagen)*.* Putting BamHI and HindIII restriction sites at amino and carboxyl ends, the construct was synthesized (GENEray Biotechnology) and subcloned in pET28a (Novagen Inc., Madison, Wis). The recombinant plasmid was transformed to competent cells and colony PCR and construct digestion were employed for cloning validation.

### Protein expression and extraction

For recombinant BMP-2 expression, the transformants were grown in LB culture media containing 15 *μg/ml* kanamycin until OD_600_=1 followed by induction with 0.2 *mM* IPTG for 6 *hr* at both 25 and 37°*C*. The harvested cells were lysed by cell lytic buffer (20 *mM* PBS, 500 *mM* NaCl, 1 *mM* EDTA, 100 *μg/ml* Lysozyme, Benzonase 80 *u/ml*, 0.1% Triton X100) and centrifuged at 14000 *g* for 30 *min*. The supernatant contained total soluble proteins which were applied to a nickel-charged affinity resin column (Ni-NTA Agarose, Qiagen 30210) and his-tag affinity was purified with imidazole buffers (300 *mM* NaCl, 50 *mM* phosphate buffer pH=8.0 and 50 to 300 *mM* imidazole) for his-tag affinity purification. The remaining pellet containing inclusion bodies was resuspended in wash buffer (20 *mM* PBS, 1 *mM* EDTA, 2% Triton X100) and centrifuged at 26000 *g* for15 *min* and then solubilized in inclusion body dissolving buffer (6M GuHcl, 0.1 *M* Tris Hcl pH=8.5, 1 *mM* EDTA, 50 *mM* DTT) and incubated for 2 *hr* at room temperature. The protein solution was then centrifuged at 14000 *g* for15 *min* and DTT was removed by dialysis against 5 *M* GuHCl, 0.1 *M* Tris Hcl, pH=3.5 overnight. Finally, a Bradford assay was run for protein content estimation.

### Protein refolding and purification

BMP-2 refolding was carried out by dilution of protein in refolding buffer (100 *mM* Tris/HCl pH=8.3, 5 *mM* EDTA, 250 *mM* NaCl, 0.5 *M* L-arginine) to decrease protein concentration and prevent aggregation. Two oxidoreductase systems (reduced glutathione/ oxidized glutathione and Cysteine/Cystine) and different refolding methods were used to analyze disulfide bond formation as well as protein dimerization (Table 1). BMP-2 refolding was started by gradual addition of denatured protein to pre-cooled refolding buffer until the concentration fell below 0.2 *mg/ml* while stirring at 4°*C* for 24 *hr*. The protein concentration increased to 1 *mg/ml* by cross flow filtration (Amicon ultra centrifugal filter) and redox system components were removed by dialysis against urea 6 *M*, 0.1 *M* Tris/HCl and 5 *mm* EDTA, pH=6. Dimeric protein was eluted by heparin column (Heparin sepharose ^™^ 6 Fast Flow, GEHealthcare, 17-0998-01) at 0.3 and 0.7 *M* NaCl and purified protein was dialyzed against 20 *mM* ammonium acetate buffer, pH=4.8 and lyophilized.

**Table 1.** Protein refolding method

**Method one**		**Method two**
Dilution in refolding buffer containing oxidoreductase system/incubation for 48 *hr*		Dilution in refolding buffer containing 0.1/0.1 *mM* reduced/oxidized oxidoreductase system, incubation up to 48 *hr* Combining and incubating with 5 *mM* /0.5 *mM* GSH/GSSG-pretreated BMP-2 for two hours and dialysis
*Oxidoreductase used system:*		*Oxidoreductase used system:*
**1. GSH/GSSG or 2. Cystein/Cystine**		**1. GSH/GSSG and 2. GSH/GSSG**
A: 2 *mM* /0.2 *mM*	C: 2 *mM* /0.2 *mM*	0.1 *mM* /0.1 *mM* and 0.5 *mM* /5 *mM*
B: 5 *mM* /0.5 *mM*	D: 5 *mM* /0.5 *mM*	

### SDS-PAGE and western blotting

Recombinant BMP-2 expression and refolding pattern were analyzed by non-reducing 12% SDS-PAGE. Separated proteins were electrophoretically transferred to nitrocellulose membranes by semi-dry blotting using a Dunn carbonate transfer buffer (10 *mM* NaCHO_3_, 3 *mM* Na2CO_3_, 20% methanol). The membranes were blocked for 2 *hr* using western blocker solution (Sigma, W0138) and incubated overnight at 4°*C*, with anti-Histag (1:30000, Penta-His Antibody mouse monoclonal Qiagene, 1008288) as a primary antibody. The blots were washed three times with washing buffer (PBS-Tween-20, 0.05%) and incubated with the peroxidase-conjugated secondary rabbit anti mouse antibody (sigma A9044) for one hour at room temperature. The blots were visualized using ECL detection reagents (Sigma, CPS-1-120) and the films were scanned with a densitometer (GS-800, Bio-Rad).

### Biological activity test of recombinant BMP-2

Biological activity of BMP-2 was measured by evaluation of its ability to increase the alkaline phosphatase activity (ALK) of a chondrogenic mouse embryonic carcinoma cell line, ATDC-5 15. The ATDC5 cell line was purchased from the RIKEN Cell Bank (RCB-0565) and a positive control was purchased from R&D company (cat no: 355-BM). ALK activity of BMP-2 induced cell line was tested using Alkaline Phosphatase Yellow Liquid Substrate System for ELISA, containing p-nitrophenyl phosphate (pNPP) (Sigma P7998).

### Cell culture

ATDC-5 cells were cultured in Dulbecco’s modified eagle’s medium DMEM-F12 (Invitrogen, 105 21331-020) supplemented with 5% Fetal Bovine Serum (FBS; Gibco Inc. Canada) and 50 *mg/ml* Non-Essential Amino Acid (NEAA) (Gibco Inc. Canada) without any antibiotic.

### Assay medium

A Serum Free Medium (SFM) was prepared for assay with RPMI 1640 (Gibco Lot no: 1668798) as a basal medium supplemented with NAEE and 0.1% bovine serum albumin.

### Assay for alkaline phosphatase activity

Briefly, SFM contained serial dilutions of recombinant BMP2 and commercial R&D BMP2 (0–500 *ng/ ml*) was provided in a 96 well flat-bottom plate and ATDC-5 cells were plated and incubated for 5 days at 37°*C* in a humidified, 5% CO_2_ atmosphere. After 5 days of culturing, cells were washed with PBS and enzyme activity was assayed by pNPP -a substrate in alkaline phosphatase assay buffer- and then plate was incubated at 37*°C* for 20 *min*. Next, the absorbance was measured at 405 *nm* with microplate reader.

## Results

### Analysis of cloning process

**Figure 1.**
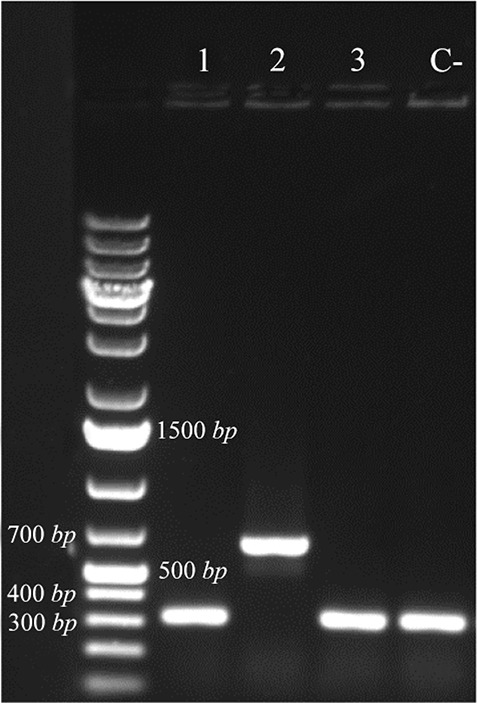
Colony PCR confirmation of recombinant plasmid by T7 primers, analyzed by 1% agarose gel electrophoresis. Lane 2 exhibited amplification in desired size. Lane C- shows amplification without desired sequence.

**Figure 2.**
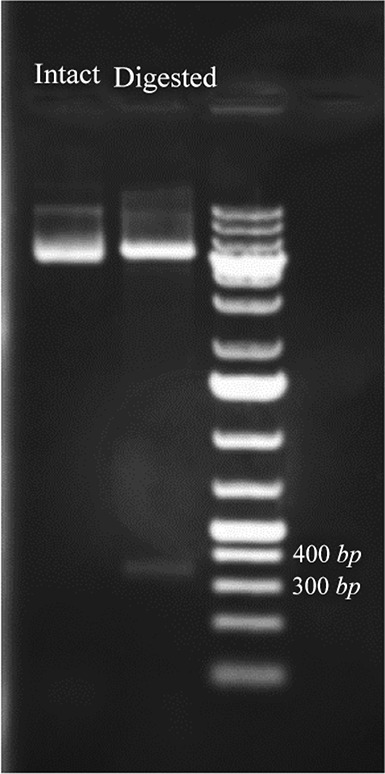
Restriction enzyme digestion of intact pET28 plasmid and recombinant plasmid which released the cloned BMP-2 insert. The plasmid DNA is analyzed by 1% agarose gel an 1 *kb* DNA ladder was used as a DNA size marker.

Synthetic coding sequence of BMP-2 which was restriction enzyme digested of pGH plasmid was sub-cloned into pET28 plasmid. After transformation of competent cells with recombinant plasmid, several colonies were selected on antibiotic plate for further analyses. Colony PCR using T7 primers (Figure 1) as well as restriction enzyme digestion of recombinant plasmid (Figure 2) amplified and released expected product sizes.

### Identification of recombinant BMP-2 protein

**Figure 3.**
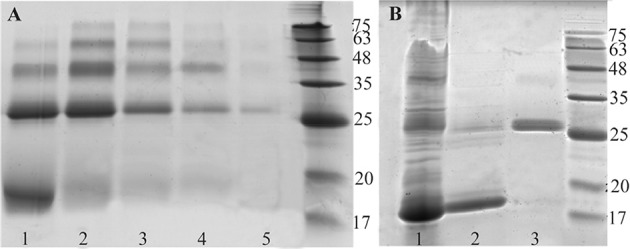
A) Nickle charged affinity resin purification of refolded BMP-2, using different concentrations of imidazole in elution buffer. Lanes 1 to 5; 30, 50, 80, 150 and 300 *mM* imidazole, respectively. B) Refolded and purified BMP-2 analysis using non-reduced 12% SDS-PAGE. Lane 1: refolded but unpurified protein. Lane 2 and 3: monomeric and dimeric forms of BMP-2 purified by heparin affinity method.

SDS-PAGE banding pattern analysis of proteins extracted from 37°*C* cultured *E. coli* showed that the recombinant BMP-2 was exclusively expressed as an insoluble fraction and accumulated in cytoplasm as inclusion bodies. Inclusion body extracted proteins exhibited a sharp band between 17 to 20 *kDa*, corresponding to monomeric chain of BMP-2 (Figure 3). Similar result was also obtained following protein expression at 25°*C* (data not shown).

Recombinant BMP-2 refolding results were assessed *via* non-reducing SDS-PAGE (Figure 3). Protein dimer formation in the refolding process accounts for approximately 40–50% at best.

**Figure 4.**
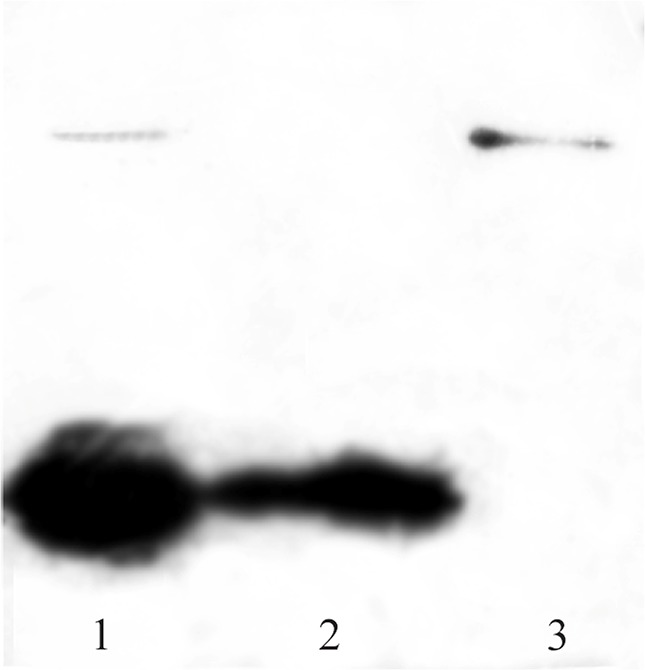
Western blot analysis of purified BMP-2, separated by 12% SDS-PAGE and transferred to nitrocellulose membrane by semi-dry electroblotting. Lane 1: unpurified protein, lane 2: monomeric protein and lane 3: dimeric form of BMP-2.

Western blot analysis (Figure 4) using anti-HisTag which strongly reacted with N-terminal histidine tag of recombinant BMP-2 protein, confirmed that migrated bands between 17 to 20 *kDa* and near 25 *kDa* are related to the monomeric and dimeric forms of the protein, respectively.

### Evaluation of recombinant protein refolding process

**Figure 5.**
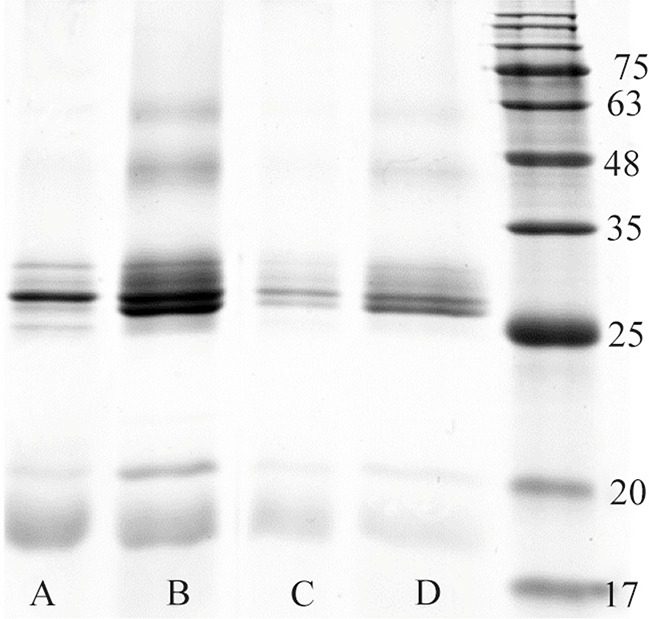
One-stage refolding of BMP-2 using refolding buffer containing cysteine/cystine (A & B) or GSH/GSSG (C & D) oxidore-ductase systems. Lane A and C: 2/0.2 *mM*, lane B and D: 5/0.5 *mM* reduced/oxidized components’ ratio.

In order to analyze the efficiency of disulfide bond formation in BMP-2 protein, two oxidoreductase systems in refolding buffer were used for refolding process (Table 1). Figure 5 illustrates electrophoretic mobility pattern of heparin affinity purified BMP-2 which was refolded by different oxidoreductase systems. A ratio of 10/1 of reduced/oxidized components was used for method one refolding process of the recombinant protein (Table 1).

The electrophoretic mobility of recombinant BMP-2 which was refolded by method two protocol (Table 1) is represented in figure 4. In this method, the unfolded protein was diluted and incubated up to 48 *hr* in refolding buffer containing 0.1/0.1 *mM* GSH/GSSG that catalyzes intra chain disulfide bond formation. Next, the solution was combined and incubated with equal volume/concentration of 5 *mM*/0.5 *mM* pretreated GSH/GSSG (product of method one) for two hours and then dialyzed. Figure 3 depicts electrophoretic pattern of refolded dimeric BMP-2 compared with that of monomeric fraction which are both eluted by 0.3 and 0.7 *M* NaCl, respectively.

### Analysis of protein purification methods

Refolded BMP-2 was purified using two methods, histidine tag affinity purification and heparin affinity chromatography. His-tag affinity purification included two essential elements.

First, N-terminal histidine tag of recombinant protein was provided by expression vector and expressed as a fusion protein with BMP-2. Second, there was nickel-charged affinity resin column for protein purification.

Different concentrations of imidazole were used in elution buffer for affinity purification of BMP-2. However, no significant differences were observed in monomer/dimer separation (data not shown). Heparin affinity purification of refolded protein is presented in figure 3. Protein purification using elution buffers containing 0.3 and 0.7 *M* NaCl showed the best results in monomer/dimer separation.

### Biological activity of recombinant BMP-2

**Figure 6.**
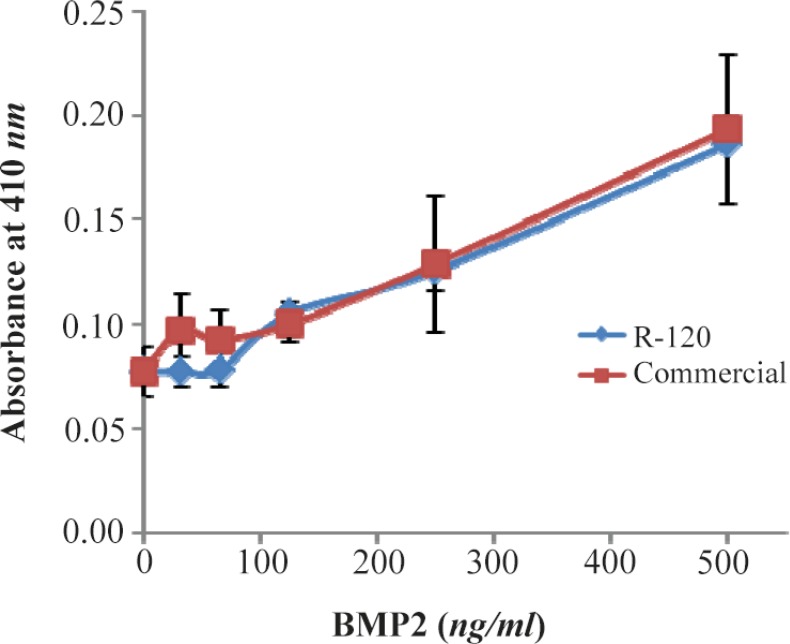
Biological activity assay of recombinant BMP-2 expressed in bacterial inclusion body (R-120) in comparison with that of commercially available eukaryotic expressed protein. Absorbance at 410 nanometer was measured as a function of ALK activity for ATDC5 cells, following the induction by recombinant BMP-2.

The biological activity assay indicated that the refolded recombinant BMP-2 increased the ALK activity of ATDC-5 cell line as much as that of commercially available eukaryotic protein (Figure 6). Increasing the concentration of refolded BMP-2 applied onto ATDC-5 cell line resulted in induction of ALK activity in a dose-dependent manner. This is a practical assay for measurement of BMP-2 activity that can be performed with high-accuracy.

## Discussion

Bacterial expression of BMP-2 resulted in production of inclusion body and therefore, protein aggregation in cytoplasm. The aggregated protein can be solubilized and extracted from inclusion body under high concentration of urea and GuHCl. Native structure of protein can be restored by decreasing urea/GuHCl concentrations and replacing them with compounds which prevent protein aggregation and promote proper folding. In the next step, these compounds should be removed gradually and protein is solubilized in appropriate buffers, capable of maintaining its active structure. Cysteine-rich proteins require a proper oxidoreductase system for disulfide bond formation or rearrangement of incorrectly formed bonds.

In this study, synthetic coding sequence of BMP-2 protein was restriction digested and subcloned in pET28a plasmid and finally transformed in Origami™ strain of *E. coli.* Verification of the insert and its orientation was approved by colony PCR amplification and restriction digestion analysis.

SDS-PAGE analysis of recombinant BMP-2 expression confirmed a high level of protein, aggregated as inclusion bodies and entirely not present in soluble fraction, in spite of decreasing IPTG induction temperature below 25°*C*. In order to restore native structure and function of GuHCl solubilized BMP-2, two different protein renaturation experiments were run. GSH/ GSSG and Cysteine/Cyctine oxidoreductase systems as well as two different protocols were employed for BMP-2 refolding process. There was no significant difference between two oxidoreductase systems in terms of dimeric structure improvement of BMP-2. On the other hand, increasing the concentration of either oxidoreductase components resulted in dimeric structure enhancement. However, the multimeric structure of BMP-2, which is the result of protein misfolding, simultaneously increases with rising in oxidoreductase components’ concentration (Figure 5).

These misfolded multimeric structures have been reported in similar studies 13,16. It was concluded that there was a positive relationship between interchain disulfide bond formation and increase in concentration of oxidoreductase systems’ components. However, no considerable biological activity of refolded protein was observed following the increase in oxidoreductase system concentration (data not shown).

In an attempt to decrease multimeric structure and at the same time increase purified dimeric protein as well as biological activity, a different refolding procedure was designed in addition to heparin affinity purification. First, the unfolded BMP-2 was diluted gradually in protein refolding buffer containing 0.1 *mM* /0.1 *mM* of GSH/GSSG for up to 48 *hr* and then dialyzed. Second, the dialyzed protein was pooled with an equal volume of BMP-2 pretreated with 5 *mM* /0.5 *mM* of GSH/GSSG and incubated for two hours. Our hypothesis was that the refolding of BMP-2 under this mild condition (0.1 *mM* /0.1 *mM* GSH/GSSG) results in correct formation of intrachain disulfide bonds and therefore monomeric renaturation. Pooling with 0.5 *mM* /5 *mM* of GSH/GSSG pretreated BMP-2-which is oxidized rather than being in reduced state-resulted in improvement of protein dimerization and thus activity. Finally, the monomeric and misfolded multimeric structures of BMP-2 were successfully eluted with high purity using a heparin affinity purification column (Figure 3).

## Conclusion

In view of well-established methods available for protein refolding, our study presented a reproducible, practical and cost-effective method for BMP-2 growth factor production in *E. coli* bacterial system which is comparable with eukaryotic systems in terms of biological activity and stability.

## References

[1] Wang RN Green J Wang Z Deng Y Qiao M Peabody M Bone morphogenetic protein (BMP) signaling in development and human diseases. Genes Dis 2014;1(1):87–105. 25401122 10.1016/j.gendis.2014.07.005 PMC4232216

[2] Wu M Chen G Li YP. TGF-β and BMP signaling in osteoblast, skeletal development, and bone formation, homeostasis and disease. Bone Res 2016;4:16009. 10.1038/boneres.2016.9 PMC4985055 27563484

[3] Carreira AC Zambuzzi WF Rossi MC Astorino Filho R Sogayar MC Granjeiro JM. Bone morphogenetic proteins: promising molecules for bone healing, bioengineering, and regenerative medicine. Vitam Horm 2015;99: 293–322. 26279381 10.1016/bs.vh.2015.06.002

[4] Kuo MM Nguyen PH Jeon YH Kim S Yoon SM Choe S. MB109 as bioactive human bone morphogenetic protein-9 refolded and purified from E. coli inclusion bodies. Microb Cell Fact 2014;13(1):29. 24559319 10.1186/1475-2859-13-29 PMC3936849

[5] Vallejo LF Brokelmann M Marten S Trappe S Cabrera-Crespo J Hoffmann A Renaturation and purification of bone morphogenetic protein-2 produced as inclusion bodies in high-cell-density cultures of recombinant Escherichia coli. J Biotechnol 2002;94(2):185–194. 11796171 10.1016/s0168-1656(01)00425-4

[6] Vallejo LF Rinas U. Folding and dimerization kinetics of bone morphogenetic protein-2, a member of the transforming growth factor-beta family. FEBS J 2013;280(1):83–92. 23122408 10.1111/febs.12051

[7] Chen J Liu Y Li X Wang Y Ding H Ma G Cooperative effects of urea and L-arginine on protein refolding. Protein Expr Purif 2009;66(1):82–90. 19233287 10.1016/j.pep.2009.02.004

[8] Tsumoto K Umetsu M Kumagai I Ejima D Philo JS Arakawa T. Role of arginine in protein refolding, solubilization, and purification. Biotechnol Prog 2004;20(5):1301–1308. 15458311 10.1021/bp0498793

[9] Samuel D Kumar TK Ganesh G Jayaraman G Yang PW Chang MM Proline inhibits aggregation during protein refolding. Protein Sci 2000;9(2):344–352. 10716186 10.1110/ps.9.2.344 PMC2144545

[10] Han B Hall FL Nimni ME. Refolding of a recombinant collagen-targeted TGF-beta2 fusion protein expressed in Escherichia coli. Protein Expr Purif 1997;11(2):169–178. 9367813 10.1006/prep.1997.0784

[11] Kim YV Gasparian ME Bocharov EV Chertkova RV Tkach EN Dolgikh DA New strategy for high-level expression and purification of biologically active monomeric TGF-beta1/C77S in Escherichia coli. Mol Biotechnol 2015;57(2):160–171. 25370824 10.1007/s12033-014-9812-7

[12] Vallejo LF Rinas U. Optimized procedure for renaturation of recombinant human bone morphogenetic protein-2 at high protein concentration. Biotechnol Bioeng 2004;85(6):601–609. 14966801 10.1002/bit.10906

[13] Sharapova NE Kotnova AP Galushkina ZM Lavrova NV Poletaeva NN Tukhvatulin AE [Production of the recombinant human bone morphogenetic protein-2 in Escherichia coli and testing of its biological activity in vitro and in vivo]. Mol Biol (Mosk) 2010;44(6):1036–1044. Russian. 21290825

[14] Zhang Y Ma Y Yang M Min S Yao J Zhu L. Expression, purification, and refolding of a recombinant human bone morphogenetic protein 2 in vitro. Protein Expr Purif 2011;75(2):155–160. 20691269 10.1016/j.pep.2010.07.014

[15] Nakamura K Shirai T Morishita S Uchida S Saeki-Miura K Makishima F. p38 mitogen-activated protein kinase functionally contributes to chondrogenesis induced by growth/differentiation factor-5 in ATDC5 cells. Exp Cell Res 1999;250(2):351–363. 10413589 10.1006/excr.1999.4535

[16] Long S Truong L Bennett K Phillips A Wong-Staal F Ma H. Expression, purification, and renaturation of bone morphogenetic protein-2 from Escherichia coli. Protein Expr Purif 2006;46(2):374–378. 16298141 10.1016/j.pep.2005.09.025

